# Toll-Like Receptors in *Leishmania* Infections: Guardians or Promoters?

**DOI:** 10.1155/2012/930257

**Published:** 2012-03-15

**Authors:** Marilia S. Faria, Flavia C. G. Reis, Ana Paula C. A. Lima

**Affiliations:** Instituto de Biofisica Carlos Chagas Filho, Centro de Ciências da Saúde, Universidade Federal do Rio de Janeiro, Bloco G, CCS, Ilha do Fundão, Cidade Universitária, Rio de Janeiro 21941-902, RJ, Brazil

## Abstract

Protozoa of the genus *Leishmania* cause a wide variety of pathologies ranging from self-healing skin lesions to visceral damage, depending on the parasite species. The outcome of infection depends on the quality of the adaptive immune response, which is determined by parasite factors and the host genetic background. Innate responses, resulting in the generation of mediators with anti-leishmanial activity, contribute to parasite control and help the development of efficient adaptive responses. Among those, the potential contribution of members of the Toll-like receptors (TLRs) family in the control of *Leishmania* infections started to be investigated about a decade ago. Although most studies appoint a protective role for TLRs, there is growing evidence that in some cases, TLRs facilitate infection. This review highlights recent advances in TLR function during *Leishmania* infections and discusses their potential role in restraining parasite growth versus yielding disease.

## 1. Introduction

Infections with parasitic protozoa have been a long-term health issue in the tropical and subtropical regions of the globe. Among those, diseases caused by infections with microbes of the *Leishmania* genus are one of the most wide-spread infirmities. *Leishmania* parasites are transmitted to the vertebrate host by the sand fly bite that injects the infective metacyclic forms under the skin. The flagellated parasites are rapidly engulfed by phagocytic cells either resident or recruited to the wound site (i.e., neutrophils, macrophages and dendritic cells), reviewed in [1]. While the passage through neutrophils is believed to be transient, serving as a temporary safe hideaway, the parasites are able to establish productive infections in macrophages, where they differentiate to amastigotes and replicate inside the parasitophorous vacuole. The pathology resulting from the infection is related to the parasite species that can either induce cutaneous (i.e., *L. major*, *L. mexicana, L. guyanensis*), mucocutaneous (i.e., *L. amazonensis*, *L. braziliensis*), or visceral leishmaniasis (*L. donovani*, *L. chagasi*). The outcomes of infections are complex, depending not only on the parasite species but also on the immune status of the host. As an example, although *L. amazonensis* is primarily associated with cutaneous manifestations, it can also provoke mucocutaneous and/or diffuse cutaneous leishmaniasis. Infections with *L. panamensis* or *L. braziliensis* can generate a persistent hyperinflammatory response, where a mixed T-helper 1 (Th1)/T-helper 2 (Th2) response is observed, leading to nonresolving lesions in humans [2, 3]. *L. guyanensis* can also migrate away from the primary infection site, generating distal secondary lesions similar to those observed with *L. braziliensis* [4].

Cutaneous leishmaniasis comprises self-healing skin ulcers in immunocompetent individuals, however, parasite persistence remains, helping to maintain protective immunity [5]. Mucocutaneous leishmaniasis involves parasitic dissemination to the nasopharyngeal area, leading to destructive secondary lesions. It is characterized by a persistent inflammatory response associated with increased expression of proinflammatory mediators that are crucial for the recruitment of cells to the site of infection [6]. About 5 to 10% of individuals asymptomatic or with resolved cutaneous lesions may develop mucocutaneous lesions [7, 8]. Although the development of a type-1 immune response is crucial for the control of parasites, unresolved inflammation can derive from lack of an appropriate modulation of those responses in the case of tegumentary leishmaniasis (reviewed in [9]). On the other hand, the progression of visceral leishmaniasis is often accompanied by a decay in the type-1 response [10]. In this scenario, increasing immunosuppression contributes to disease progression not only due to the presence of immunoregulatory cytokines but also because of partial destruction of lymphoid tissues [11, 12].

The mouse model for cutaneous leishmaniasis caused by *L. major* has been largely explored over the years, providing a solid body of data defining the immunological mechanisms involved in mounting innate and adaptive protective responses (reviewed in [13]). However, those findings cannot be readily extrapolated for models of infections by other *Leishmania* species, rendering it indispensable to investigate the parameters of immunological responses for each combination of parasite species, host cell, and/or animal model for the disease. Although adaptive immunity is essential for the resolution of infection, there is growing evidence that innate mechanisms make an important contribution to the antiparasitic defenses.

Innate responses develop after the initial sensing of invading microbes, leading to the production of effector molecules that contribute to contain initial infection and to mount the subsequent adaptive immune response [14]. The early immune reaction against *L. major*, *L. braziliensis,* and *L. infantum* during experimental infections has been analyzed in detail, revealing, for example, differential susceptibility to nitric oxide (NO) and reactive oxygen species (ROS) in phagocytes located at distinct organs. While the production of NO is required for the leishmanicidal activity against *L. major* [15] and *L. braziliensis* [16] in the skin of infected mice, it is dispensable in the spleen and mildly important in the lymph node. Although NO was believed to be important to the control of *L. donovani* in the liver and spleen of mice [17], it was later established that neither iNOS nor NADPH oxidase (phox) is essential to restrict parasite replication in the liver [18, 19]. The relative importance of such mediators has been recently covered by Liese and coworkers [14] and will not be explored in this review.

The potential contribution of Toll-like receptors in fighting parasitic infections has gained attention in the last decade. We will cover the main findings regarding the interplay between Toll-mediated responses and *Leishmania* infections.

## 2. Toll-Like Receptors (TLRs) and *Leishmania*

Toll-like receptors (TLRs) are hallmarks of cellular receptors that recognize pathogen-associated molecules and participate in innate responses to infections (reviewed in [10]). There are currently 13 mammalian TLRs described, while TLRs 1–9 are functionally conserved between humans and mice, TLR10 seems to be functional in humans but divergent at the C-terminus in the mouse, rendering it inoperant. TLR11 is functional in the mouse but truncated in humans. The conserved TLRs can be divided into extracellular: TLR1-2, TLR4-6, and TLR11 [20] or intracellular: TLR3, TLR7-9 and TLR13 [21], and those receptors recognize specific groups of ligands either at the cell surface or in the endosomal compartment, respectively [22]. Each TLR detects distinct sets of molecules from viruses, bacteria, fungi, and parasites, and upon binding, they recruit different adaptor proteins such as MyD88 or TRIF [22]. TLRs initiate innate responses in a variety of ways, leading to the production of inflammatory cytokines by macrophages and different subtypes of dendritic cells (DCs) and of type I interferons (IFN) by inflammatory monocytes, macrophages, and DCs [22]. Neutrophils also express the majority of TLR family members and several coreceptors but lack intracellular TLR3 and TLR7 (reviewed in [23]). In those cells, TLR activation often leads to the generation of reactive oxygen species (ROS), cytokine production, increased cellular survival, receptor expression, and phagocytosis [24].

 The microbial molecules recognized by TLRs are conserved polymers, such as bacterial lipopolysaccharides (LPSs), peptidoglycans, unmethylated bacterial DNA, and double-strand viral RNA, among others. Since protozoans lack most of these structures, TLRs must recognize other groups of molecules in order to sense those microbes. The recognition of *Trypanosoma cruzi* tGPI (glicosylphosphatidylinositol anchor) by TLR2 [35] and of glycoinositolphospholipids by TLR4 [25], of *Plasmodium* hemozoin by TLR9 [26], and of the profilin-like molecule of *Toxoplasma gondii* by TLR11 [30] are such examples.

The phagocytosis of *Leishmania* by macrophages, contrary to the observed with other pathogens, is marked by the absence of many proinflammatory cytokines [27]. Furthermore, infected macrophages become unresponsive to subsequent challenges with the TLR4 ligand LPS, a feature that is associated with parasite phosphoglycans [28, 36]. Since TLR recognition is often associated with the production of proinflammatory cytokines and with the generation of additional effector molecules, it is unquestionably important to determine the implications of TLR activation during *Leishmania* infections. A few *Leishmania*-derived molecules have been reported to activate TLRs, and the majority of the studies to date focused on the activation of TLR2, TLR4, and TLR9.

### 2.1. TLR2 and MyD88

Initial experiments suggesting that *Leishmania* induces TLR-mediated responses came from studies using cells lacking the adaptor molecule MyD88 [29]. Those analyses showed that *L. major* activates the promoter region of IL-1*α*, but not of IL-6, IL-8, or IL-10, through MyD88-dependent pathway in macrophages [29]. It was then reported that MyD88-dependent pathways are required for the development of the protective IL-12-mediated Th1 response against the *L. major* in C57BL6 resistant mice, since MyD88^−/−^mice infected with *L. major* developed a nonprotective Th2 response [37]. The animals had enlarged nonhealing lesions and low IL-12 plasma levels suggesting that TLR-mediated responses were important to develop effective antiparasite immunity. At the same time, it was revealed that the higher susceptibility of MyD88^−/−^ mice to *L. major* infections correlated with elevated levels of IL-4, despite the lack of ulcerating lesions [38]. *L. donovani*, *L. braziliensis*, *L. major,* and *L. mexicana* induce the maturation of DCs *in vivo* and the observation that DC maturation was attenuated in MyD88^−/−^ mice infected with *L. donovani* implied the involvement of TLRs in DC maturation and T-cell priming [39]. Although the studies using MyD88-deficient mice suggested that TLRs influence the adaptive immune response during *Leishmania* infections, they did not offer undisputable evidence for the participation of TLRs, mainly because MyD88 can also function as an adaptor protein to the IL-1 receptor [40]. Furthermore, there are pathways downstream of TLR3 and TLR4 which are independent of MyD88, such as the activation of TLR4 through IRF3 leading to the production of type-1 interferons [41]. Therefore, it became crucial to prove the direct involvement of TLRs for the responses observed in the infection models described above.

The direct activation of TLR2 by *Leishmania* components was subsequently reported. Purified *L. major* lipophosphoglycan (LPG) induced the upregulation and stimulation of TLR2 on human NK cells, with additional enhancement of TNF-*α* and IFN-*γ* [42]. LPGs of *L. major*, *L. mexicana*, *L. aethiopica*, and *L. tropica* were defined as TLR2 ligands in studies using murine macrophages, although the stimulation with *L. tropica* LPG was only marginal [34]. At this point, the possibility that species- or strain-specific structural differences in the phosphoglycan chain could contribute to receptor recognition or activation was raised. Those studies revealed that the induction of TNF-*α* synthesis by *L. major* LPG required the presence of the lipid anchor and of functional MyD88. Intriguingly, LPG also induced the expression of suppressors of the cytokine signaling family proteins SOCS-1 and SOCS-3 [34]. The finding that negative modulators of cytokine synthesis were also induced by LPG indicated that TLR2 stimulation by *L. major* can lead to both positive and negative inflammatory signals. The fact that SOCS1 itself directly downmodulates TLR4 signaling pathways [43] illustrates how the initial stimulation of TLR2 by *L. major* can ultimately lead to the attenuation of further TLR responses.

**Figure 1:**
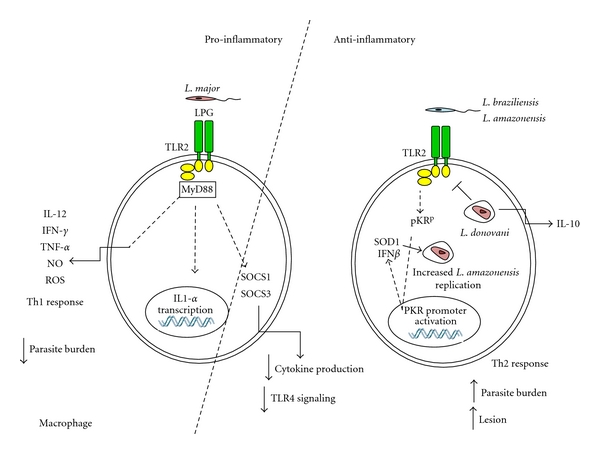
Model for the activation of TLR2 by *Leishmania sp.*, leading to a proinflammatory (left), or anti-inflammatory arm (centre and right). In early studies, *L. major* stimulated the transcription of IL1*α* through a MyD88-dependent pathway, but not the IL1*α* production at the protein level, suggesting the triggering of anti-inflammatory stimuli negatively controlling IL1-*α* production [22]. LPG or additional undefined TLR2 ligands promote the synthesis of cytokines, nitric oxide (NO), and reactive oxygen species (ROS) that are related to parasite killing and to the development of a protective Th1 response, through MyD-88-dependent pathways [22–29]. *L. major* LPG also induces the production of suppressors of the cytokine signaling family proteins SOCS-1 and SOCS-3 [26], whose activities are associated with diminished cytokine production and prevention of TLR4 signaling [30]. Lack of TLR2 increased the resistance to infections with *L. braziliensis* [31] and *L. amazonensis *[32] decreased lesion formation and parasite burdens, suggesting that TLR2 is required for disease promotion. In RAW macrophages, the infection with *L. amazonensis* promotes the phosphorylation of PKR and the activation of the PKR promoter and enhances the synthesis of both PKR and of type 1-IFNs, and those events require TLR2 [33]. The levels of SOD1 expression are elevated in association with PKR activation and IFN*β* production, resulting in increased parasite replication [33]. *L. donovani* downmodulates TLR2 responses in macrophages by inhibiting MAPp38 kinase, leading to IL10 production [34]. The dashed lines indicate that intermediate steps of the pathways are either not identified or not represented in the figure.

More recently, it was shown that LPG stimulates cytokine production by human peripheral blood mononuclear cells via TLR2 as well [31]. Although cellular LPG isolated from promastigotes is structurally similar to soluble LPG present in culture supernatants, they differ in the average number of phosphorylated oligosaccharide repeat units and in glycan composition [32]. In the above-mentioned study, while both forms of LPG induced the production of ROS in a TLR-2-dependent manner, Th1-promoting cytokines were induced solely by soluble LPG, leading to the proposal that the first encounter and recognition of *L. major* by membrane-derived LPG after interaction with TLR2 provides a cytokine milieu for consequent Th2 differentiation [31]. Later on, the same authors provided evidence that the induction of NO production by macrophage cell lines was achieved with both LPG forms, and was dependent on TLR2, but not on TLR4 signaling [44]. More recently, synthetic oligosaccharides based on the LPG structure were shown to induce IL-12 and Th1 responses *in vivo* through TLR2, corroborating that TLR2 stimulation by *Leishmania* LPG potentiates the inflammatory responses in mice [33]. Those findings assign a protective role for TLR2 and MyD88 during *L. major* infection, in particular, TLR2 seems required to mount an effective Th1 response (Figure 1, left pannel).

The consequences of TLR2 activation to the control of leishmaniasis was further verified using the TLR2 ligand, arabinosylated lipoarabinomannan (Ara-LAM), in a Balb/c model of visceral leishmaniasis [45]. Ara-LAM induced the expression of TLR2 in macrophages infected with *L. donovani in vitro*, which was accompanied by the production of NO and of proinflammatory cytokines. Mice pre-treated with Ara-LAM and subsequently challenged with *L. donovani *showed around 80% reduction of infection in the liver and the spleen, paralleled by a strong Th1 response. However, one should take care at interpreting those results since Ara-LAM could exert its effect *in vivo* through additional mechanisms besides TLR2 activation.

The parasite can also modulate the expression of TLRs, interfering with their availability and/or the subsequent quality of the responses mediated by those receptors. For example, two antigens isolated from *L. donovani* amastigotes were shown to upregulate TLR2 expression in RAW264 macrophages [49]. The 65 and 98 kDa proteins induced elevated levels of MAPK p38, as well as its phosphorylation in relation to that of ERK1/2, leading the authors to conclude that they induced the activation of TLR signaling proteins. However, one should interpret those conclusions with caution, specific controls for the activation of TLR2 (i.e., neutralizing antibodies or cells derived from knock-out mice) were lacking in this study. Furthermore, the contribution of minor nonproteinaceous contaminants in the preparations cannot be excluded considering that the antigens were isolated by elution from SDS-PAGE gels and were not further purified. In another study, while verifying the expression of TLRs during the infection of Balb/c mice with *L. chagasi*, attempts were made to correlate upregulation of TLR expression with a potential role for those receptors in generating inflammatory versus anti-inflammatory responses [50]. The authors observed upregulation of TLR2 and TLR4 from day 1 to day 28 postinfection, and increased expression correlated with higher mRNA levels for TNF*α*, IL-17, IL-10, and TGF*β* during early infection. On the other hand, there was an inverse correlation between the expression of TLR2-4 and that of IL-12 or IFN-*γ*, and parasite load, leading to the proposal that those TLRs are involved in the recognition of the parasite during visceral leishmaniasis. However, while most of those studies evaluated the induction of cytokine mRNA and of mRNA for TLRs, they did not provide direct evidence that TLRs are required for the induction of cytokine synthesis. Increased expression of TLRs might have occurred as a consequence of the inflammatory environment in the spleen, making it difficult to evaluate to what extent the activation of TLRs is implicated in those findings.

*L. panamensis* was found to induce upregulation of TLR1, TLR2, TLR3, and TLR4 in human primary macrophages [51]. The activities of TLR4 and TLR3 correlated with TNF-*α* secretion and the leishmanicidal activity of those macrophages [51]. A global analysis of gene expression in IFN-*γ*-treated THP-1 macrophages infected with *L. major* showed that genes related to TLR signaling are also differentially expressed upon infection [46], revealing how the parasite could interfere with the capacity of the host cell to respond via TLR-mediated routes. *L. major* can also induce the expression of TLR2, TLR7, and TLR9 in polymorphic nuclear cells (PMNs) from C57BL6 mice [52], evidencing that infection provokes alterations in the levels of several TLRs in multiple cells of the innate system.

Additional parasite molecules were implicated in TLR2 activation. The *L. infantum* protein related to the silent information regulator 2 (SIR2) family leads to the proliferation of activated B lymphocytes, causing increased expression of major histocompatibility complex (MHC) II and the costimulatory molecules CD40 and CD86, in a TLR2-dependent fashion [53]. The maturation of DCs induced by SIR2, accompanied by the secretion of IL12 and TNF, was also dependent on TLR2, while the activation of macrophages still took place in the absence of TLR2.

Other studies suggest that *Leishmania* downregulates TLR2-mediated responses and attempted to dissect some of the downstream molecular pathways involved therein. The infection of human THP1-derived macrophages by *L. donovani in vitro* suppresses TLR2 and TLR4-stimulated IL-12 release, with an increase in IL-10 production, through parasite-dependent contact [54]. Parasites were shown to negatively modulate the TLR2-stimulated signaling pathway by suppressing MAPKp38 phosphorylation and activating extracellular regulated kinase (ERK)1/2 phosphorylation [54]. It was also reported that *L. donovani*, *L. mexicana*, and *L. major* exploit the macrophage tyrosine phosphatase SHP-1 to inactivate kinases involved in TLR signaling [55]. The infection of bone-marrow-derived macrophages *in vitro* leads to IRAK inactivation, impairing LPS-mediated activation as well as macrophage function [55].

 Studies using TLR2-deficient mice revealed a somewhat contrasting scenarium (Figure 1, right pannel). TLR2 was necessary for the development of lesions in mice infected with *L. braziliensis* [56]. While MyD88^−/−^ mice developed larger and prolonged lesions compared to those in control mice, the lack of TLR2 resulted in enhanced DC activation and increased IL-12 production after infection. *L. braziliensis*-infected TLR2-deficient DCs were more competent in priming naïve CD4 T cells *in vitro*, correlating with increased IFN-*γ* production *in vivo* and enhanced resistance to infection. More recently, it was reported that TLR2-deficient mice are also less susceptible to the infection with *L. amazonensis* than the wild-type C57BL6 counterpart, as evidenced by lower parasite burden and reduced recruitment of inflammatory cells in the first weeks of infection [57]. At the same time, it was described that the activation of the protein kinase PKR during the infection of macrophages by *L. amazonensis* is crucial for parasite survival [58]. Finally, The engagement of PKR and the subsequent production of type-1 IFNs and of superoxide dismutase (SOD-1) were necessary for effective parasite growth and dependent on TLR2, indicating that this TLR is required for the successful infection of macrophages by *L. amazonensis* [47]. Those findings suggest that TLR2 plays a role in facilitating the establishment of the disease, depending on the *Leishmania* species in question.

### 2.2. TLR4

**Figure 2:**
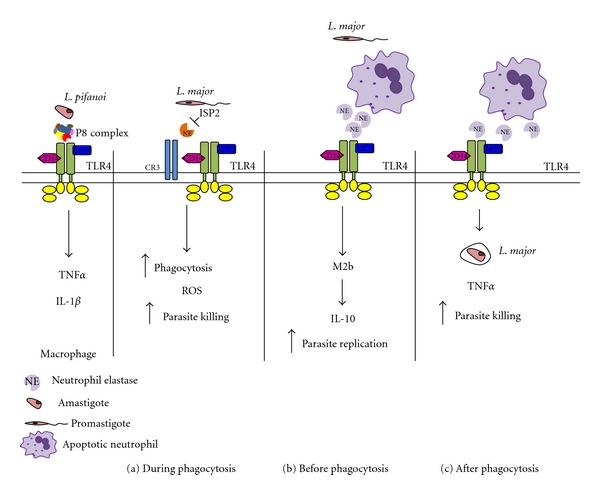
Model for the activation of TLR4 in different settings of *Leishmania* infections. *L. pifanoi* amastigote-derived P8 glycolipid complex stimulates TLR4 in macrophages in an MD2, CD14, and MyD88-dependent manner, leading to the production of proinflammatory cytokines (left panel) [46]. Microenvironments during the activation of TLR4 in macrophages of C57BL6 mice by neutrophil elastase (NE): in (a), during the phagocytosis of *L. major* promastigotes, CR3, TLR4, and NE at the surface of macrophages increase parasite uptake but lead to ROS production and to partial parasite elimination within 24 h [47], the control of NE activity by parasite ISP2 prevents TLR4 activation and protects parasite from intracellular killing [47, 48]; in (b), macrophages remove apoptotic neutrophils by phagocytosis and acquire a M2b phenotype, leading to IL10 production and increased permissiveness to parasite growth; in (c), macrophages are infected by *L. major* and subsequently interact with apoptotic neutrophils, resulting in the activation of TLR4 by NE and in the production of TNF that promotes parasite killing.

 Besides the direct activation of TLRs by parasite molecules, the engagement of those receptors indirectly by nonparasite ligands during phagocytosis could influence the outcome of infection. Studies using TLR4^−/−^mice indicated that this receptor plays a protective role in *L. major* infections [48]. The authors observed diminished parasite load at the skin lesions of infected mice at the initial stages of infection, that is, 24 h, and found increased parasite survival in host cells from TLR4-deficient mice, which correlated with a higher activity of arginase [48]. The same group subsequently showed that the lack of TLR4 results in increased parasite growth during both the innate and adaptive phase of the immune response and in delayed healing of the cutaneous lesions [59]. However, TLR4 does not seem important to define the range of chemokines produced in the skin or in the draining lymph nodes of mice infected with *L. major* [60]. In agreement with a protective role for TLR4, it was described that the lack of SLAM, a cell surface receptor of macrophages that regulates TLR4-transduced signals, increased the susceptibility of mice to *L. major* infections [61]. Importantly, this phenotype was attributed to defective macrophage function, suggesting that the activation of host cell TLR4 contributes to the control of parasite growth *in vivo*. The first potential TLR4 ligand present in *Leishmania* was described a few years ago in *L. pifanoi* amastigotes and consists of a proteoglycolipid complex (P8) composed of a cysteine and serine metalloprotease, host-derived ApoE, and four glycolipids [62, 63]. Stimulation of macrophages with P8 or its isolated glycolipids provoked the synthesis of a range of proinflammatory cytokines, including IL1*β* and TNF*α* and the responses to intact P8 were dependent on TLR4, MD2, CD14, and MyD88 [62] (Figure 2, left). However, the exact molecular entity serving as the TLR4 ligand in P8 still remains to be determined. More recently, TLR4 was implicated in the mechanism underlying the inhibition of IL-12 production in LPS-treated macrophages infected with *L. mexicana*. Metacyclic promastigotes were found to greatly increase the phosphorylation of the three major MAP kinases, ERK, p38, and JNK, in a manner dependent on TLR4 but not on TLR2 [64]. Parasites prolonged the induction of iNOS or COX-2 expression in LPS-stimulated macrophages, enhanced PGE2 and NO production, and increased the expression of arginase-1. The induction of iNOS, COX-2, and arginase-1 were also dependent on TLR4, supporting the hypothesis that this TLR plays an anti-inflammatory role in macrophages during *L. mexicana* infections, ultimately preventing IL-12 production.

On the other hand, the anti-leishmanial role of TLR4 might be exerted by multiple downstream effector molecules. For example, macrophages of the mouse strain SPRE/Ei, which is resistant to LPS, have normal MyD88-mediated signaling pathways but are defective in the production of type-1 IFNs [65]. Those mice were found to be highly susceptible to *L. major*, a feature that was attributed to the poor induction of IFN*β*-dependent genes. These observations suggest that type-1 IFNs pose as effectors that, through a paracrine/autocrine loop, contribute to parasite control downstream of TLR4 stimuli. The cross-talk between TLR4 and additional receptors should also be taken into consideration when viewing anti-leishmanial properties attributed to this TLR. For example, in macrophages, the stimulation of the glucocorticoid receptor by progesterone downmodulates TLR4-induced NO and IL-12 production and reduces the killing of *L. donovani* by activated macrophages [66] Likewise, complement-derived C5a negatively regulates TLR4-induced IL-12, IL-23, and IL-27 in macrophages, leading to decreased Th1 responses *in vivo* [67]. It was found that Th1- enhanced immunity in C5a-receptor^−/−^mice confers protection against an *L. major* challenge [67], exemplifying how other receptors of innate immunity might affect susceptibility to leishmania infections through the modulation of TLR responses.

The engagement of TLRs during the interaction between different cells of the innate system can also dictate the fate of parasites in infected macrophages. It was reported that apoptotic neutrophils from C57B6 mice induce the production of TNF-*α* by *L. major*-infected macrophages* in vitro*, leading to parasite elimination [68]. Later on, the activation of macrophage TLR4 by the neutrophil-derived serine protease, neutrophil elastase (NE), was proposed as the underlying mechanism leading to parasite death [69] (Figure 2, scheme c). More recently, the phagocytosis of apoptotic neutrophils by bone-marrow-derived macrophages in a proinflammatory context *in vitro* was found to induce a stable M2b phenotype, rendering those macrophages more permissive to the replication of *L. major* [70]. The induction of the M2b phenotype, correlating with higher IL10 levels and a Th2-type response, was dependent on TLR4 and NE activities, suggesting that the engagement of TLR4 might be beneficial to the parasite at later stages of infection (Figure 2, scheme b). Indeed, rechallenge of those macrophages with LPS promoted parasite growth and the treatment of C57B6 mice with apoptotic neutrophils 3 days before infection increased parasite burdens, demonstrating how TLR4-mediated signals can contribute to create permissive niches for parasite replication at later stages [58].

We have recently described that NE present at the surface of murine macrophages can also activate TLR4 during the phagocytosis of *L. major*, without requirement of neutrophils [71]. Wild-type *L. major* is able to control NE activity, at least in part, through its endogenous protease inhibitor structurally similar to bacterial ecotins, named ISP2 (inhibitor of serine peptidases) [72]. Parasites lacking ISP2 promptly engage macrophage TLR4 due to uncontrolled NE activity [71] (Figure 2, scheme a). Furthermore, we found cooperativity between TLR4 and CD11b, a subunit of the complement type 3 receptor (CR3), in facilitating the uptake of *L. major* by macrophages. TLR4 activation led to the production of ROS, provoking partial elimination of intracellular parasites a few hours after internalization [71]. We found that the cooperativity between CD11b and TLR4 to enhance the phagocytosis of parasites was strictly dependent on the activity of NE, setting another example of how multiple components of innate immunity can act concertedly to improve the defense against invading parasites. The ability of *Leishmania* to promote the cross-talk between those receptors might exacerbate TLR4 responses in light of the finding that optimal signaling through TLR4 requires multiple cell surface associated molecules such as CD11b and HSP90 [73]. We observed that early engagement of TLR4 can influence how macrophages respond to *L. major* in at least two fronts: (i) first, by promoting enhanced phagocytosis and early killing of promastigotes (i.e., 24 hrs), and (ii) secondly, by restraining the subsequent growth of amastigotes in the following 2–4 days. Those observations suggest that signals mediated through TLR4 at the parasitophorous vacuole could affect the microenvironment surrounding the parasite, controlling sustained intracellular growth.

Recently, a screen of TLR4 mutations in patients with cutaneous leishmaniasis revealed that certain genotypes were largely favoured in individuals with chronic or acute disease, as compared to asymptomatic donors or noninfected individuals, leading to the proposal that TLR4 polymorphism may lead to increased susceptibility or severity of the disease [74]. Although the consequences of TLR activation in macrophages as a result of *Leishmania* infections are being widely investigated, we are still lacking information on how TLRs might affect the interaction of the parasites with neutrophils. The mobilization of TLRs in neutrophils, in particular that of TLR4, is known to induce a wide range of responses. Of interest, IRAK4 is crucial for the exocytosis of neutrophil secretory granules induced by TLR4 [75]. In view of the findings that NE affects the outcome of parasite growth in macrophages [69–71], the stimulation of TLR4 in neutrophils could regulate the levels of secreted NE, influencing parasite burden in macrophages.

### 2.3. TLR9 and TLR3

Studies aiming at improving the therapy for *L. major *infections using recombinant IL-18 pointed to a potential involvement of the CpG-TLR9 pathway in the protective antiparasite response. While promoting rIL18 expression in infected mice through gene gun delivery, the authors observed a synergistic role of the plasmid vector with rIL18, which was dependent of CpG motifs [76]. The induction of proinflammatory cytokines, in particular of IL-12, by antigen-presenting cells was found to be dependent on TLR9 activation by CpG, indicating that TLR9 stimulation could lead to protective immunity against the parasite. In attempting to define which TLRs were involved in the increased susceptibility of MyD88-deficient mice to *Leishmania* infections, Liese and coworkers showed that TLR9 was required to induce IL-12 in bone-marrow-derived DCs by either *L. major* or its DNA [77]. Furthermore, they observed that TLR9^−/−^ mice exhibited more severe skin lesions and higher parasite burdens as compared to C57B6 controls, coupled to a transient increase in IL-4, IL-13, and arginase. Even though there was an increase in Th2 cytokines, they did not observe alterations in the levels of IFN*γ* in the draining lymph node, concluding that deficiency in TLR9 does not influence the quality of the Th1 response [77]. The same group reported that plasmacytoid DCs responded to *L. infantum* by secreting IFNs *α* and *β* and IL-12 in a TLR9-dependent manner and that NK-induced cytotoxicity was abolished in TLR9^−/−^mice [78]. Those results were the first to link TLR9, IL-12, and DCs to the activation of NK cells during visceral leishmaniasis. The protective role of NK cells in murine leishmaniasis is now proven to be strictly dependent on IL-12 secreted by myeloid DCs in response to *Leishmania* via TLR9 (reviewed in [14]).

**Figure 3:**
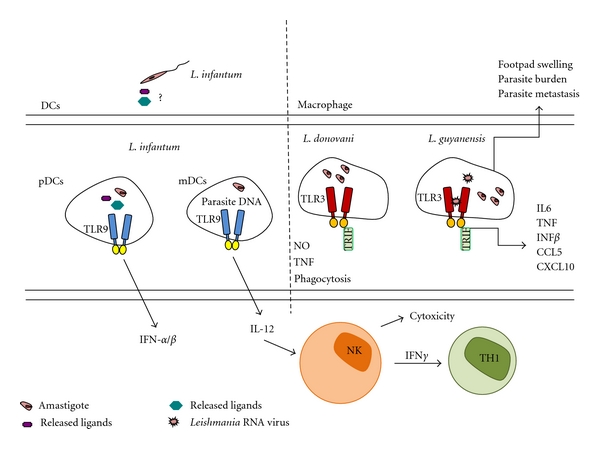
Proposed models for the activation of intracellular TLR9 and TLR3 by *Leishmania*. TLR9 of plasmocytoid DCs (pDCs) can be activated by putative TLR9 ligands secreted by *L. infantum* promastigotes and taken up by endocytosis or in endosomal compartments. TLR9 in myeloid DCs (mDCs) can be activated by *L. infantum* DNA after parasite phagocytosis and destruction in the endosomal compartment, leading to the activation of NK cells and protective Th1 responses [4, 63, 64]. TLR3 is required for the production of TNF, NO and the phagocytosis of *L. donovani* by macrophages, suggesting a protective role for infection [67]. Intracellular TLR3 is activated in a TRIF-dependent pathway in macrophages infected with metastatic *L. guyanensis*, by double-stranded RNA from the *Leishmania* virus LRV1 [68]. This leads to the production of proinflammatory cytokines and chemokines, as well as type1-IFNs. Despite the inflammatory response, TLR3 is required for the effective development of footpad swelling, for significant parasite burden and its dissemination in infected hamsters, suggesting that TLR3 activation facilitates disease pathology by parasites of the *Leishmania Viannia* subgenus.

The involvement of TLR9 in controlling *L. major* infection was further investigated in the following studies. Lesion progression and parasite burden were higher in TLR9^−/−^ mice as compared to C57BL/6 controls, which was concomitant with a transient inhibition of a Th1 response [79]. The authors showed that the activation of bone-marrow-derived DCs, or of DCs isolated from the spleen, by *L. major* or by parasite DNA, followed by IFN-*γ* production by CD4 T cells, was abolished by treatment with DNAse or by alkalinization of endosomal compartments with chloroquine, supporting a role for TLR9 in DC activation by the parasite [79] (Figure 3, left panel). Those findings caused some controversy as to the impact of TLR9 deficiency in attenuating the Th1 response [80]. Since in both studies there were delays in the healing of skin lesions in TLR9^−/−^ mice, it is feasible that the Th1 response is at least partially compromised in those mice.

The recognition of *Leishmania* by TLR3 in endosomal compartments was brought up a few years ago. Experiments using RNA interference provided evidence that in IFN-*γ* primed macrophages, TLR3 is required for the production of NO and TNF-*α* induced by infection with *L. donovani*, besides contributing to parasite phagocytosis [81] (Figure 3, right panel). Since *Leishmania* does not contain double-stranded RNA, the standard ligand for TLR3, the origin of the stimulatory factor of TLR3 remained an open question. More recently, an elegant work from Ives and coworkers shed light on this matter, providing evidence that RNA from viruses present in *L. guyanensis* serve as a source of TLR agonists, promoting inflammatory cytokines and chemokines [82] (Figure 3, right panel). The elevated cytokine production was dependent on the TLR3-TRIF pathway, and augmented via MyD88-transduced signals. Intriguingly, those responses rendered mice more susceptible to infection, as observed by increased swelling and parasite burden, together with more pronounced metastasis. Those findings add to the growing list of examples where the subversion of TLR responses by *Leishmania* serves to promote infection instead of playing a protective role.

Additional factors can further influence TLR activation indirectly, affecting the outcome of adaptive immunity. For example, it was found that dyslipidemia inhibits TLR-induced production of IL-12, IL-6, and TNF-*α*, as well as upregulation of costimulatory molecules by CD8*α* ^−^ DCs, *in vivo*, leading to impaired Th1 and enhanced Th2 responses. Such dysfunction compromised host resistance to *L. major*, revealing that a dyslipidemic microenvironment can interfere with DC responses to *Leishmania* through TLRs [83].

### 2.4. TLRs Stimulation in Vaccination and Immunotherapy

 The use of TLR activators as adjuvants in formulations to improve the efficacy of experimental vaccination against *Leishmania* has also been largely explored. Even before the knowledge that TLR stimulation could play a role in the defense against *Leishmania*, CpG oligodeoxynucleotides (ODNs) were tested as an adjuvant in the immunization of mice with *L. major*-soluble antigen aiming at inducing a protective Th1 response, leading to improved survival [84]. This observation supported the proposal that TLR agonists could serve as cost-effective alternatives in vaccine formulations against leishmaniasis. Along those lines, the formulation of multisubunit recombinant vaccines with the TLR4 agonist, monophosphoryl lipid A, elicited protective immunity against *L. major* challenge in Balb/c mice, an approach that could serve as an alternative to the use of recombinant IL12 [85]. An analogue of lipid A was subsequently used as a TLR4 agonist in experimental vaccination with *L. amazonensis* antigens and was proven beneficial for both immunoprophylaxis and immunotherapy, associated mainly with increased levels of IL12 and IFN*γ* [86]. CpG (ODN) were throughoutly tested as adjuvants in experimental vaccinations to induce protective responses to *L. donovani* [87, 88], or to *L. major* in Balb/c mice, when coencapsulated with antigens in liposomes [89]. CpG also protected Balb/c mice immunized with ribosomal proteins against a second challenge with *L. major*, being effective in providing long-term immunity [90, 91]. Finally, Cpg ODN improved the clinical outcome of immunized rhesus macaques infected with *L. major*, supporting the hypothesis that TLR9 stimulation might improve the efficacy of vaccination against cutaneous leishmaniasis [92]. Using a combined approach of costimulation of different TLRs, the immunogenicity of recombinant antigens from *L. donovani* (iron superoxide dismutase B1 and peroxidoxin 4) was greatly improved by immunization in conjunction with TLR9 or TLR4 agonists, resulting in a Th1-type response in Balb/c mice [93]. The use of ligands for TLR7 and/or TLR8 (imiquimod and the related R848 compound) in the immunization of Balb/c mice with crude *L. major* antigen induced a Th1 response and was protective against subsequent challenges [94].

 More recently, the formulation of a defined polyprotein anti-*Leishmania* vaccine candidate in conjunction with TLR4 or TLR9 agonists was evaluated as an immunotherapeutical treatment in a mouse model of cutaneous leishmaniasis [95]. A strong effective T cell response was observed during disease, followed by cured lesions and reduced parasite burden upon immunization with the combination of both agonists, suggesting that TLR synergy may serve as a tool for the treatment of parasite infection. TLR2 stimulation was also proposed as an alternative to revert the loss of CD8 function in patients with diffuse cutaneous leishmaniasis [96]. It was found that, in patients infected with *L. mexicana* who develop diffuse lesions, the reduced cytotoxicity and proliferation of CD8 cells was typical of cellular exhaustion and could be restored *in vitro* by stimulation with TLR2 agonists, including *Leishmania* LPG [96]. The design of CD8 activators based on TLR2 activation could be beneficial in reverting the observed in chronic patients with diffuse lesions. More recently, a more sophisticated vaccination approach was attempted in order to promote protective responses against *L. panamensis* in a murine model of chronic disease [97]. The strategy of prime-boost, consisting of the priming using a single antigen and heterologous DNA, was combined with the addition of a TLR2/1 agonist (PAM3CSK4) as an adjuvant, leading to an effective protection against *L. pananemensis* infections [97]. Furthermore, they provided evidence that TLR2 stimulation during priming is essential for the elevation of CD4 and CD8 memory T cell responses and reduction of IL-13 and IL-10 levels, which were required for protection. Those findings were a great step forward in the design of simple and cost-effective vaccines against *Leishmania* (*Viannia*) species that are refractory to commonly used strategies to other *Leishmania* species. It also offers an alternative for DNA-based vaccination schemes in cases where the CD8 T cell activation is crucial.

## 3. Concluding Remarks

The activation of several members of the TLR family of receptors is observed during the infections of isolated cells or in experimental infections of animals with different *Leishmania* species. The consequences of such activation are complex and depend on the nature of the TLR, the cell type, the parasite species, and the timing in which those events occur. Although studies using TLR-deficient animals offered the initial picture of how those receptors can act in the protection or promotion of disease, the underlying molecular mechanisms are still obscure. In particular, it is indispensable to identify the parasite factors that contribute to TLR activation and/or negatively regulate their responses in the individual cell types and to evaluate to what extent TLR activation in each of those cells contributes to pathology. While most of the earlier studies focused on the requirement of TLRs for cytokine synthesis and for the development of adaptive immunity, the studies on if and how those receptors contribute to the biogenesis of the parasitophorous vacuole and to the control of parasite intracellular growth are still largely missing. TLR activation has emerged as a promising alternative to improve the efficacy of vaccination with parasite antigens and may also act synergistically with other components during immunotherapy. However, a more detailed study of the consequences of such strategies must be addressed before the combined activation of TLRs can be explored in the future in the treatment of patients with more severe and diffuse lesions.
